# Physicochemical, Rheology, and Mid-Infrared Spectroscopy Techniques for the Characterization of Artisanal and Industrial Maroilles Cheeses

**DOI:** 10.3390/foods13193086

**Published:** 2024-09-27

**Authors:** Gaoussou Karamoko, Romdhane Karoui

**Affiliations:** University Artois, University Lille, University Littoral Côte d’Opale, University Picardie Jules Verne, University Liège, INRAE, Junia, UMR-T 1158, BioEcoAgro, F-62300 Lens, France; gaoussou.karamoko@univ-artois.fr

**Keywords:** Maroilles cheeses, rheology, MIR, physicochemical, secondary structure

## Abstract

The investigation of the central and external zones of ten industrial and artisanal Maroilles cheeses showed differences in their physicochemical parameters, namely fat, pH, moisture content, ash, and color. This difference significantly impacted the rheological properties of the investigated cheeses, which depended on the protein network englobing lipid and water and its interaction with the other components. Overall, Maroilles cheeses had an elastic-like behavior, with the central zones exhibiting the greatest viscoelastic modules (G′ and G″). The mid-infrared (MIR) spectra highlighted the presence of lipids, proteins, and sugars. A significant difference in α-helix and β-sheet levels in the central zones was noted between artisanal and industrial Maroilles cheeses. It is suggested that the difference between artisanal and industrial Maroilles cheeses observed at the macroscopic level, due to the cheese-making procedure and ripening stage, affects the structure at the molecular level, which can be determined by MIR spectroscopy. This trend was confirmed by the FDA when applied to the MIR spectra, since 96.67% correct classification was noted between artisanal and industrial cheeses. The present study indicates that MIR spectroscopy can be used successfully to study Maroilles cheese samples belonging to different production chains.

## 1. Introduction

For a long time, soft, semi-hard, and hard cheeses have played a significant role in human nutrition [1]. Maroilles is a soft cheese with a washed rind, made in the Hauts-de-France region (France), particularly in the Thiérache region, and labeled as a protected designation of origin: PDO [2]. In 2019, the consumption of cheese in France was 26.8 kg per person per year [3]. In the same year, 417,935 tons of soft cheeses were produced, including 4441 tons of Maroilles cheeses [3].

Maroilles cheese could be produced from raw milk (artisanal cheese) and pasteurized milk (industrial cheese). The ripening stage of Maroilles cheese varied between 3 and 5 weeks depending on the size (quarter, mignon, sorbais, and big) and scale (artisanal and industrial). During the ripening stage, Maroilles is washed 2–3 times/week with a brine of 30 g/L and red ferment composed of a mixture of microorganisms (*Geotrichum candidum* and *Kluyveryomyces lactis*). These microorganisms are of technological interest and are widely used during the ripening stage. They are considered ripening ferments and are naturally associated with milk, although in low proportions. *Geotrichum candidum* is a dimorphic yeast responsible for the evolution of the cheese surface during ripening [4], while *Kluyveromyces lactis* is capable of consuming lactose and fermenting it under both anaerobic and aerobic conditions [5].

The texture of Maroilles cheeses is heterogeneous between the surface and the center zones due to the development of red ferment at the surface. This difference induced some modification in the level of proteolysis, lipolysis, glycolysis, and so on, provoking differences in the structure between the surface and center zones. Different techniques were used to evaluate the quality of soft cheeses, namely Camembert cheese [6,7] “Lokum kivaminda Beyaz peynir” Turkish soft white cheese [8]; Spanish ewe’s milk PDO soft cheeses named “Torta del Casar” and “Queso de la Serena” [9]; and Italian soft cheeses [10]. However, only limited studies were dedicated to the Maroilles cheeses where the (i) pigment fingerprints of the rinds of Maroilles, including French PDO red-smear ripened soft cheeses Epoisses and Mont d’Or was determined as well as the bacteria causing this pigmentation [11]; (ii) denaturing high-performance liquid chromatography was used for the identification of yeast in red smear cheese surface including the Maroilles, Munster, and Livarot [12]; (iii) MALDI-TOF spectrometry was utilized for the identification of lactic acid bacteria in Maroilles cheeses [2]; and (iv) physicochemical, sensory, and microbiological analyses were applied for evaluating the difference between industrial and artisanal Maroilles cheeses [13,14]. In addition, the few studies that have been performed on artisanal and/or industrial Maroilles cheeses have focused on their physicochemical, sensorial, and microbiological properties.

Given the variability of milk composition, Mid-Infrared Spectroscopy (MIR) has proven to be an essential tool for the dairy industry to provide quantitative information on milk and dairy products in terms of composition and molecular interactions. This technique has often been used to characterize cheese by investigating its authenticity and quality [15,16], determining its geographical origin [17,18], and measuring its composition [19,20]. Nevertheless, to the best knowledge of the authors, no study has addressed the impact of the physicochemical variations at the surface and center zones of Maroilles cheese on the structure determined by MIR. Therefore, the aim of the present research study was to use different analytical techniques, namely physicochemical, rheology, and MIR, for the characterization of Maroilles cheeses collected from industrial and artisanal production chains. In addition, the relationships between the protein secondary structure and the physicochemical parameters of Maroilles cheeses were investigated as a function of the sampling zones.

## 2. Materials and Methods

### 2.1. Cheese Samples

Ten types of commercial (5 artisanal and 5 industrial) Maroilles cheeses were utilized in the present study, as illustrated in Table 1. For each cheese sample, two samples were collected from the same batch. The quarter-sized Maroilles samples weighing ~ from 180 to 200 g had an average ripening time of 21 days. The samples were studied at the end of their ripening stage with a difference of a few days (3 to 4 days). Cheese samples were kept in a refrigerator before being cut at the central and external zones for MIR, rheology, pH, moisture, ash, and color measurements. Due to the limited availability of cheeses used in the present study, the central and surface zones were homogenized for lipid and protein analyses. External zone sampling was performed from 3 mm depth after the rind, while cheese heart was considered for the central zone samples. All analyses were performed in triplicates.

### 2.2. Physicochemical Analyses

Cheese samples were analyzed for total nitrogen (TN) by Kjeldahl using a factor of 6.38 as a conversion factor to determine the crude protein. The fat content was determined by the butyrometric method [17]. The pH value was measured using a digital pH meter (WTW pH 330i taschen-pH meter, WTW GmbH, Troistedt, Germany). The moisture content was determined by placing 5 g of cheese in a capsule previously dried in an oven Air concept (Firlabo, Collegien, France) at 105 °C for 24 h until a final constant weight was attained. The capsule was then transferred to a desiccator and weighed [21]. To determine the ash content, 3 g of cheese sample was weighed in a crucible and placed in a muffle furnace (Nabertherm, Lilienthal, Germany) at 600 °C for 5 h to obtain complete carbonization. The crucible was transferred to a desiccator, and after cooling, it was weighed to obtain the ash content [21].

### 2.3. Color Measurements

Color analysis was carried out using a CR-300 spectrocolorimeter (Konica Minolta Sensing Europe, Roissy-en-France, France). Measurements were performed on cheese samples using the CIE L*a*b* colorimetric system. The lightness L* varied from 0 (dark) to 100 (light), a* describing the intensity from red (+60) to green (−60), and b* describing the intensity from yellow (+60) to blue (−60).

### 2.4. Rheology Analysis

The viscoelastic analyses of cheese samples were performed from small amplitude oscillatory shear (SAOS) tests using a HAAKE Mars III rheometer (Thermo Scientific, Courtaboeuf, France) equipped with a Peltier system (TM-PE-P). The measurements were carried out using a parallel geometry P35 (35 mm diameter and 0.5 mm gap). Cheese samples in the central and external zones were gently portioned into cylindrical shapes (diameter of 35 mm and thickness of 5 mm). To prevent dehydration, the samples were placed in plastic bags until analysis. To gain insight into the network structure and viscoelastic behavior, frequency sweep experiments were performed over a range of 1 to 100 Hz, using a 0.2% strain in the linear viscoelastic region, and the storage and loss moduli (G′ and G″) were recorded. All rheological measurements were performed in triplicate at 20 °C, and the data were processed using HAAKE RheoWin software 4.86.002.

### 2.5. Mid-Infrared Spectroscopy Measurements

A Fourier transform spectrometer IRTracer-100 (Shimadzu, Duisburg, Germany) mounted with an attenuated total reflection (ATR) was used to acquire spectra of cheese samples in the range of 4000 to 900 cm^−1^ at a resolution of 4 cm^−1^. Cheese slices at the central and surface zones (8 cm length, 2 cm width, and 0.5 cm thickness) obtained using a slicer were deposited on the crystal and gently pressed with a press accessory, ensuring intimate good contact between the sample and the crystal. Before each measurement, the ZnSe crystal was recorded and used as the background. Between different cheese samples, the crystal was carefully cleaned using ethanol and ultra-pure water.

Changes in the secondary structure were obtained by applying the Fourier second derivative of the Amide I region (1700–1600 cm^−1^) using LabSolutions software (LabSolution IR 2.31, Shimadzu) to evaluate the integrated areas of β-sheet, random coil, α-helix, and β-turn peaks. The four protein secondary structures were determined according to the following assignments: 1600–1640 cm^−1^ (β- sheet), 1640–1650 cm^−1^ (random coil), 1650–1660 cm^−1^ (α-helix), and 1660–1690 cm^−1^ (β-turns) [22].

### 2.6. Statistical Analyses

To compare the samples, ANOVA was applied to each variable of the physicochemical measurements. For the MIR measurements, different pre-treatments (normalization, multiple scatter correction, and second derivative) were applied to the spectra. Principal component analysis (PCA) was applied separately to the normalized physicochemical and MIR spectra.

In the second step, factorial discriminant analysis (FDA) was performed on the 5 PCs resulting from the PCA applied to each spectral region in the MIR (3000–2800, 1700–1500, and 1500–900 cm^−1^) using the concatenation approach. The aim of this technique is to predict the membership of an individual to a pre-defined qualitative group. Four groups were defined: artisanal central cheeses, artisanal external cheeses, industrial central cheeses, and industrial external cheeses. Comparison of the assigned group to the real group is an indicator of the quality of the discrimination. Due to the low number of the investigated Maroilles cheese samples, FDA with cross-validation was applied. The same samples were used for both model calibration and prediction.

ANOVA and FDA were performed with XLSTAT 2014 (Addinsoft SARL USA, New York, NY, USA) software, while PCA was determined using MATLAB software (Matlab, Version 6.5, Release 12, The MathWorks).

## 3. Results and Discussion

### 3.1. Chemical and Color Characterization of Maroilles Cheese Samples

Protein, fat, pH, moisture, ash, and color data are reported in Table 2 (A,B). All these parameters were investigated at the central and external zones, except for protein and fat contents, where the surface and central zones were mixed for analysis.

Protein content varied from 18.94 to 24.09%, in agreement with the findings of Nacef et al. (2019b) [14], who reported values varying between 19.65 and 23.16% for the same variety of cheese. Our results indicated that the artisanal and industrial cheese samples did not show a significant difference regarding the protein content at *p* > 0.05 (Table 2 (A)), in disagreement with the findings of Nacef et al. (2019b) [14], who observed a significant difference (*p* < 0.05) between artisanal and industrial Maroilles cheese samples. One explanation could arise from the fact that out of the 10 studied cheeses in the present study, only five cheeses were common to the study conducted by Nacef et al. (2019b) [14] and ours. Regarding the fat content, artisanal Maroilles cheeses were characterized by higher levels (28.75%) compared to the industrial ones (25.13%). These results are in agreement with those of Nacef et al. (2019b) [14], who reported fat content of 28.55 and 26.77% for artisanal and industrial cheeses, respectively. This difference in fat content between traditional and artisanal cheeses could be explained by the impact of the pasteurization process, which might lead to a loss of volatile compounds and fat, as depicted by Petrus et al. (2011) [23].

For artisanal cheese, the pH values ranged from 4.96 to 6.25 and from 5.63 to 6.90, in the central and external zones, respectively. Regarding industrial cheeses, the pH is in the 5.45–5.82 and 6.36–6.74 range in the central and external zones, respectively. Industrial cheeses depicted higher values than artisanal cheeses for both the external and central zones. According to Ozbek and Guzeler (2022) [24], a high pH value of cheese is related to the pasteurization temperature. Regardless of the type of cheese, higher pH values were observed in the external zones. This result is consistent with the findings of Karoui and Dufour (2003) [6], who observed higher pH values at the surface of three retailed soft cheeses than at the center. This difference could be ascribed to the development of *Geotricum candidum* and *Kluvveryomyces lactis* microflora at the surface of the cheese during ripening, which would be responsible for the increase in pH due to the consumption of lactic acid.

For moisture content, Table 2 (A) shows a significant difference (*p* < 0.05) between cheese samples according to their type (artisanal or industrial) and location (surface or center zones). Furthermore, industrial Maroilles cheeses presented higher moisture levels than artisanal ones in both the external and central zones, in agreement with previous studies [14,25]. The moisture levels in the central zone were 49.57 and 53.88% for artisanal and industrial cheeses, respectively. Higher values were observed in the external zone, since values of 51.41 and 55.76% were noted, respectively, for artisanal and industrial cheeses. These results are in agreement with those of Karoui and Dufour (2003) [6], who reported that the dry matter content in the central zones was higher than that at the surface layers of three ripened soft cheeses. The difference in moisture content between the central and external zones of the cheeses could be due to (i) either the transfer of hydrated ions from the central part to the rind, (ii) the development of *Geotricum candidum* and *Kluvveryomyces lactis*, which ensures the absorption of water at the surface, and/or (iii) an increase in the hydration of surface proteins, resulting from the pH rise [26].

Regarding the ash content, Table 2 (A) shows that for a considered zone (central or external), higher values were observed for the industrial cheeses than for the artisanal ones. In addition, for the considered cheese type, higher values were noted in the external zone than in the central zone. Our results are in line with the findings of Le Graet et al. (1986) [27], who depicted a higher ash amount for the Beaufort cheese at the external part compared to the central part (63.4 versus 60.11%, respectively). This trend could be explained by the migration of minerals (calcium and phosphorus) from the central zone to the external zone during the ripening stage.

Table 2 (B) shows the L*, a*, and b* values of the industrial and artisanal cheese samples in the central and external zones. Overall, the results revealed that the L* parameters did not present significant differences (*p* > 0.05) between artisanal and industrial cheeses, although higher values of L* were observed for industrial cheeses. Regarding a* and b* values, no significant difference (*p* > 0.05) was observed between industrial and artisanal cheeses in the central zone, while a significant difference (*p* < 0.05) was observed in the external zone. These results are similar to those obtained by Buffa et al. (2011) [25] and Nacef et al. (2019b) [14]. The difference observed between the artisanal and industrial cheeses for a* and b* at the surface zone could be related to the treatment applied to milk (raw for artisanal versus pasteurization for industrial) and/or technological processes. Furthermore, by comparing the color of the samples investigated in the central and external zones, our results are consistent with those of Nacef et al. (2019b) [14]. It was found that the L* value decreased, while the a* and b* values increased from the central to the external zones of the Maroilles samples. This means that the central zone of Maroilles cheese was brighter, while its surface was redder and yellower.

### 3.2. Global Analysis of Physicochemical and Color Data Sets

**Figure 1:**
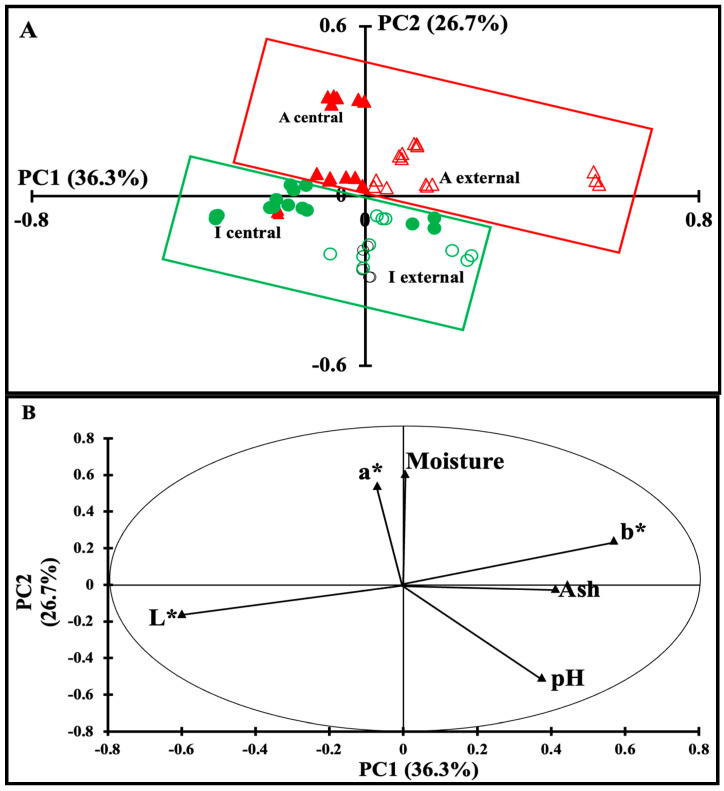
Principal component analysis (PCA) performed on the normalized physicochemical data for artisanal and industrial Maroilles cheese samples (A: Artisanal; I: Industrial). (**A**) similarity map by PC1 and 2 (A central-full triangle, A external-open triangle, I central-full circle, I external-open circle) and (**B**) correlation chart of variables.

In order to extract information from physicochemical and color data sets, multivariate statistical analyses were applied. Figure 1A shows the similarity map of the PCA performed on the normalized physicochemical and color measurements. This map, defined by PC1 and PC2, accounting for 36.3 and 26.7% of the total variance, respectively, revealed differences between Maroilles cheese samples according to their type (artisanal or industrial) and sampling (center or surface) zones. The PC1 separated cheeses cut at the external zones, which presented mostly positive scores, in contrast to those belonging to the central zones, exhibiting mostly negative scores. According to the PC2, artisanal Maroilles samples had mostly positive score values, whereas the industrial Maroilles samples presented mostly negative ones.

Based on these results, the correlation circle was studied to determine the variables responsible for discrimination between cheese samples (Figure 1B). According to PC1, the artisanal external Maroilles cheese samples were characterized by the highest values of b*, while the industrial central Maroilles cheese samples presented higher lightness (L*). PC2 indicated that high values of moisture and a* were observed for the artisanal central Maroilles samples, while the industrial external samples had the highest pH values. These results indicate that parameters such as color, pH, and moisture are responsible for the differences observed between Maroilles cheese samples, showing their heterogeneity according to cheese type and sampling zone. This is in agreement with the finding of Nacef et al. (2019b) [14], who found that the physicochemical variables allowed discrimination between the artisanal and industrial Maroilles cheeses.

### 3.3. Viscoelastic Behavior of Maroilles Cheese Samples

**Figure 2:**
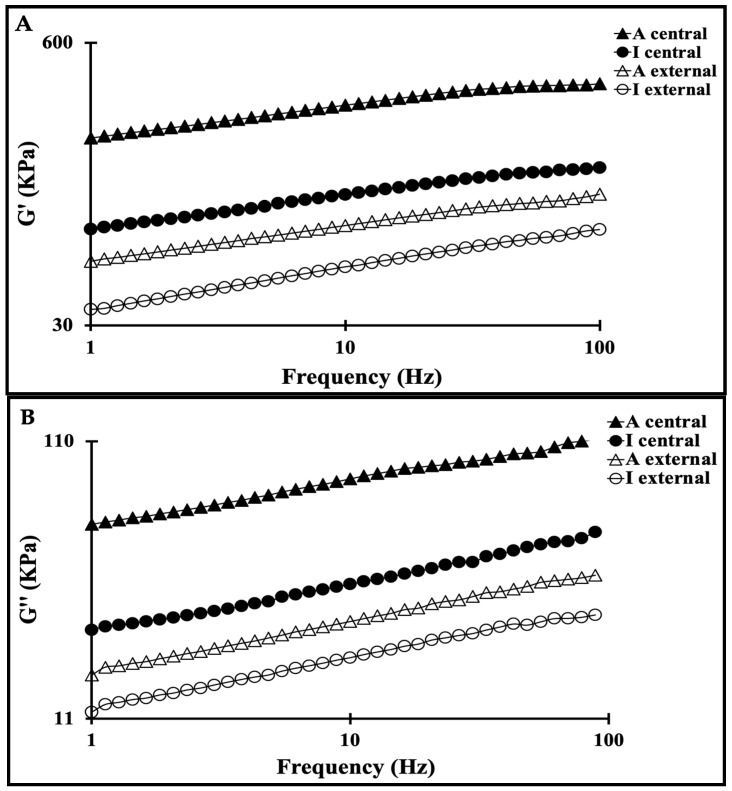
Evolution of the viscoelastic behavior G′ (**A**) and G″ (**B**) of artisanal (A) and industrial (I) Maroilles samples collected at the external and central zones (A central-full triangle, A external-open triangle, I central-full circle, I external-open circle).

The rheological behavior of Maroilles cheese was determined by applying low-strain oscillation. Due to their composition (moisture, protein, fat, and carbohydrate), cheeses exhibit a viscoelastic characteristic (both elastic and/or viscous-like behavior). As shown in Figure 2A,B, a general trend emerged for Maroilles samples. Overall, the mechanical responses of Maroilles samples revealed an elastic-like behavior within 1 to 100 Hz regardless of the sample location and cheese type. Artisanal Maroilles samples presented the highest values of G′ and G″ compared to industrial Maroilles samples. According to previous studies, the viscoelastic behavior of cheese is highly affected by several factors such as protein, fat, moisture, pH values, and type and maturity level of cheese [6,28,29,30].

It appeared that pH was the primary factor affecting the rheological properties, while dry matter seemed to have a secondary effect. Indeed, artisanal cheeses cut in the central zone having the lowest pH value presented the highest G′ and G″ values, while industrial cheeses cut at the surface presenting the highest pH value exhibited the lowest G′ and G″ values, in agreement with the findings of Karoui and Dufour (2003) [6]. The differences in the viscoelastic behavior of Maroilles cheese samples might also be explained by the fat and protein content, as highlighted by Karoui and Dufour, (2003) [6]. Indeed, the highest value of G′ and G″ could be attributed to the higher fat content of artisanal Maroilles samples compared to the industrial ones due to the fat content and the size of the fat globules in milk, which might modify the cheese rheological properties. However, it is difficult in the present study to attribute any difference in the rheological properties to protein content because, according to Table 2 (A), no significant differences (*p* > 0.05) were observed between samples, protein-protein, protein-lipid and/or protein-water interactions would impact the G′ and G″ parameters. It can be concluded that the rheological properties of cheese depend on the organization of the protein network and its interaction with other components, which vary according to milk composition, pasteurization scale, and manufacturing process.

### 3.4. Mid-Infrared Spectra of Maroilles Cheese Samples

**Figure 3:**
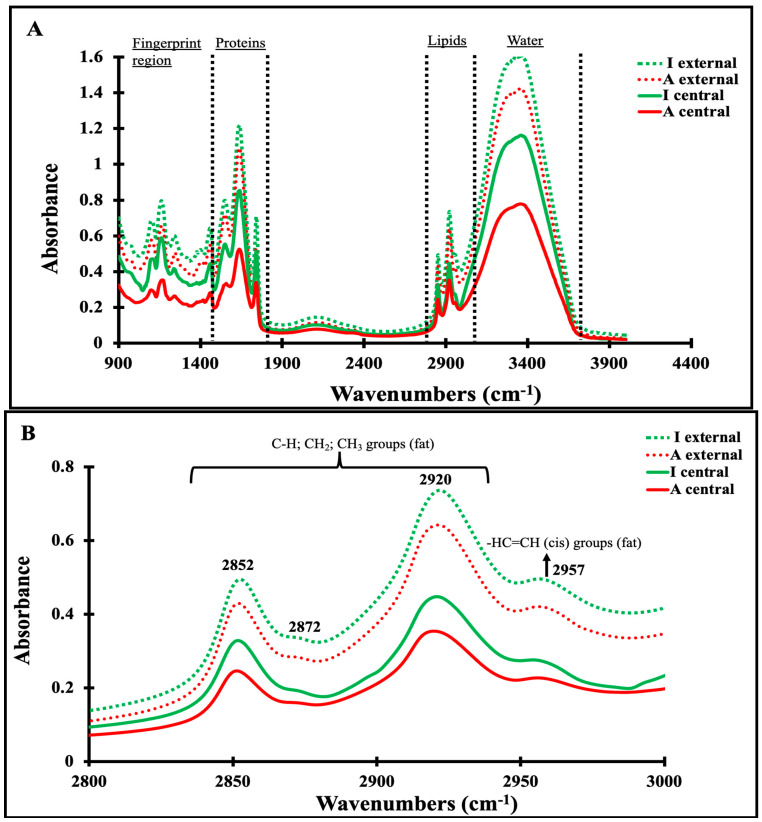

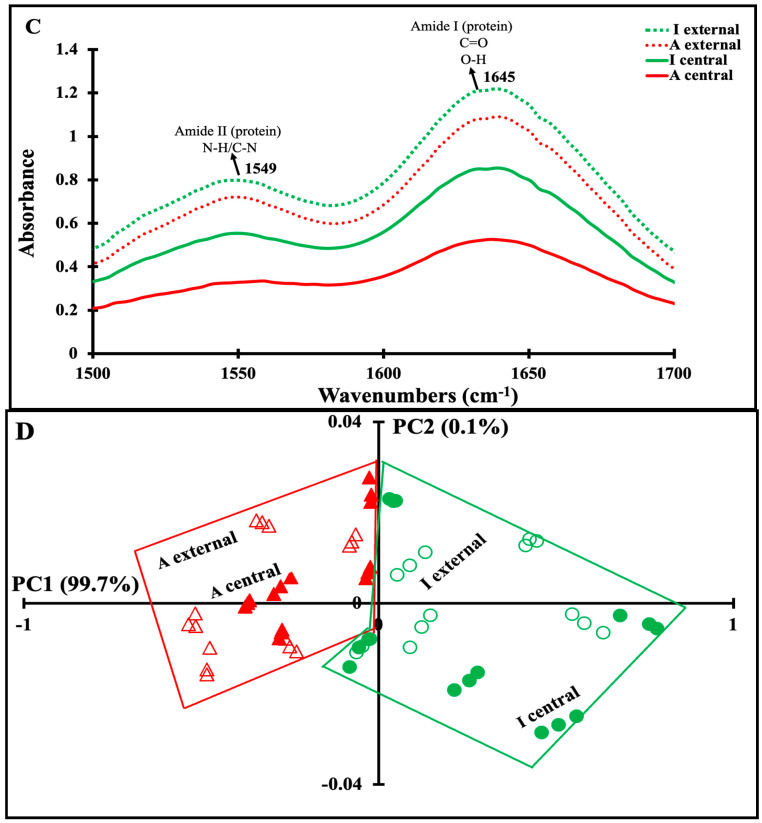

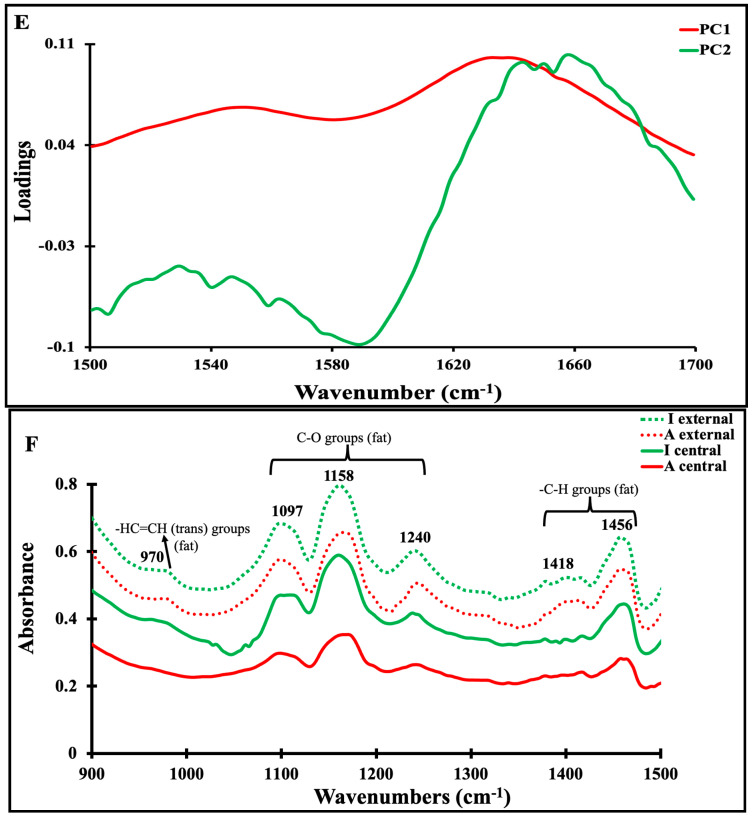
Mid-infrared analysis of artisanal (A) and industrial (I) Maroilles cheeses samples collected from the central and external zones: (**A**) 4000–900 cm^−1^; (**B**) 3000–2800 cm^−1^; (**C**) 1700–1500 cm^−1^; (**D**) PCA similarity map determined by PC1 and PC2 in the region 1700–1500 cm^−1^ (A central-full triangle, A external-open triangle, I central-full cercle, I external-open cercle); (**E**) spectral patterns 1 and 2 of PCA applied to 1700–1500 cm^−1^ and (**F**) 1500–900 cm^−1^.

Figure 3A displays the MIR spectra scanned on Maroilles cheeses, revealing common characteristic features attributable to the main components in cheese, including water, protein, lipids, and carbohydrates. MIR spectra presented some differences according to the sampling zones (external and central) and type (artisanal and industrial). The spectral profiles of Maroilles cheeses are similar to those scanned on milk, as indicated by Grewal et al. (2017) [31]. The spectra were dominated by one band ~3360 cm^−1^, which was assigned to the stretching vibration of the O–H bond present in water molecules [32]. The regions encompassing the major spectral information were studied separately: 3000–2800 cm^−1^, 1700–1500 cm^−1^, and 1500–900 cm^−1^. These regions showed some changes in the band absorbance between the investigated samples according to the cheese type and sample location.

The 3000–2800 cm^−1^ spectral region (Figure 3B) corresponds to the C–H bond of the methyl and methylene groups of fatty acids [33]. It was characterized by two predominant bands located at 2920 and 2852 cm^−1^, assigned to methylene anti-symmetric and symmetric stretching modes, respectively. Two other weak bands were observed at 2957 and 2872 cm^−1^, ascribed to the asymmetric and symmetric stretching modes of the terminal methyl group [34]. These observations are in agreement with the findings of Karoui et al. (2007) [34], who observed four bands in Gruyère PDO and L’Etivaz PDO Swiss cheese samples: two major bands located at 2919 and 2851 cm^−1^ and two weaker bands at 2955 and 2872 cm^−1^, respectively. The highest absorbance was observed for industrial cheese samples, regardless of the sample location (center or surface), suggesting a low fatty acid content. This trend could be due to the differences in the nature and content of fatty acids present in the cheese samples and also their crystallization state. It has been mentioned that the carboxyl groups of fatty acids are modified during the cheese-making process by microorganism enzymes in milk, which would be the origin of the diversity of taste found in cheeses.. This would partially explain the differences in the 3000–2800 cm^−1^ region between cheeses, as evidenced by the examples of Gruyère PDO and L’Etivaz PDO Swiss cheese samples [34] and Maroilles cheese samples in the present study.

Figure 3C presents the spectra in the 1700–1500 cm^−1^ region, characteristic of bands related to proteins that can provide information on protein-protein, protein-water, and protein-lipid interactions. The spectra of the investigated cheese samples showed two bands, as reported by Karoui et al. (2007) [34] for the Gruyère PDO and L’Etivaz PDO cheeses. The band observed at 1645 cm^−1^ was ascribed to the Amide I band based on symmetric stretching vibrations of the carbonyl functional group that is used to investigate the secondary structure of proteins. The absorption band at 1549 cm^−1^ was assigned to the Amide II bands, based on N–H bending coupled with C–N stretch vibrations [33]. In this region, significant differences were observed in the band absorbance between artisanal and industrial cheeses. The spectra of industrial cheeses exhibited the highest absorbance, regardless of the sample location. Despite their equivalent protein content (Table 2 (A)), this difference in absorbance between both kinds of cheese could be explained by a modification of the protein structure and/or the protein-lipid, protein-protein, and protein−water interactions, as well as protein interactions with the other components. Pasteurization is known to partially denature milk proteins and, therefore, alter their structure [35].

Since rheological properties and protein contents based on cheese sample location (external and central zones) were different, it was assumed that their structures at the molecular level and, as a consequence, the MIR profile at the 1700–1500 cm^−1^ region might exhibit some differences. Indeed, samples belonging to the central zones presented higher absorbance than those taken at the external zone. This might be linked to the pH difference observed between the sample locations. According to Ong et al. (2020) [36], pH change induces protein charge modification and, as a consequence, modification in protein conformation. This was confirmed by the secondary structure of the investigated cheeses (Table 3), indicating that the secondary structure of Maroilles cheeses varied according to the cheese sampling zones. Indeed, the secondary structures of artisanal and industrial cheeses were not significant in the external zones. However, in the central part, the secondary structure showed a significant variation in β-sheets and alpha helix levels between artisanal and industrial cheeses. The β-sheets content was 29.07% for industrial cheeses versus 27.29% for artisanal cheeses; the α-helix level was higher in artisanal cheeses (14.73%) compared to the industrial cheeses (13.98%). This structural modification in α-helix and β-sheet amounts agreed with the finding of Nassar et al. (2023) [35], who reported the effect of pasteurization on the α-helix and β-sheet structure levels. Moreover, the works of Tarhan and Kaya, (2021) [37] supported these findings whereby the pasteurization increased α-helix amount and led to the exposure of β-sheet and β-turn structure. However, in our study, the β-turn levels were not modified enough to obtain a significant difference between industrial and artisanal cheeses. For random coil amounts, no significant changes were also observed. It can then be concluded that only the α-helix and β-sheet structures were greatly modified during the milk pasteurization and/or ripening stage. It is known that milk proteins undergo unfolding and partial denaturation when exposed to high pasteurization temperatures, and conserve this conformational modification during the cheese ripening stage [37].

In order to extract information from MIR data, PCA was applied separately to each spectral region (1500–900, 1700–1500, and 3000–2800 cm^−1^) scanned on Maroilles cheese samples. The resultant similarity map of the PCA applied to the 1700–1500 cm^−1^ is illustrated in Figure 3D. It is defined by the principal components 1 (PC1) and 2 (PC2) that explained 99.7% and 0.1% of the total variance, respectively. Figure 3D differentiates the two groups along PC1. The first group exhibited mostly positive scores belonging to the industrial Maroilles samples, while the second group corresponded to the artisanal Maroilles samples and presented mostly negative scores. These results show that the 1700–1500 cm^−1^ region offers a clear separation of industrial Maroilles cheese from artisanal Maroilles cheese. It seems then that the 1500–1700 cm^−1^ region did not allow good separation of Maroilles samples according to their sampling zones. Based on the loading plot (Figure 3E), which reveals the contribution of each wavenumber in the score plot of the samples, it can be concluded that the Maroilles cheese separation was caused predominantly by the strongest band located at 1634 cm^−1,^ which could be ascribed to aggregated intermolecular β-sheet structures. The high absorbance for β-sheet might explain the secondary structure modification between the industrial and artisanal Maroilles cheese samples due to the pasteurization effect and storage time.

The spectra scanned in the 1500–900 cm^−1^ region, the so-called fingerprint region, are depicted in Figure 3F. This region has been used to authenticate and discriminate cheeses according to their geographic origins [7,33,34]. This results in C–O, C–C, and P=O stretch vibrations and the bending modes of O–C–H, C–C–H, and C–O–H [38]. Again, industrial cheeses exhibited higher absorbance than artisanal cheeses; in addition, for the considered type, cheese belonging to the central zone had the highest absorbance than that belonging to the external zone. The strongest bands were located at 1456, 1240, 1158, and 1097 cm^−1,^ and the weakest at 1418 and 970 cm^−1^. The 1462, 1418, and 1240 cm^−1^ bands are due to the bending vibrations of -C–H, =C–H, and -CH2-, as reported previously by Karoui et al. (2007) [34]. The 1158 and 1097 cm^−1^ bands are related to the -C–O stretching vibration. The last band is located between 1000 and 900 cm^−1^. This zone, likely related to polysaccharide vibrations [11,15,16], exhibits some characteristic peaks of fatty acids, like the obtained band at 970 cm^−1^ corresponding to CH (trans) out-of-plane deformation vibration, being reported as a peak of an unsaturated fatty acid [34]. The PCA applied to the 3000–2800 and 1500–900 cm^−1^ allowed clear discrimination between artisanal and industrial Maroilles cheeses.

**Figure 4:**
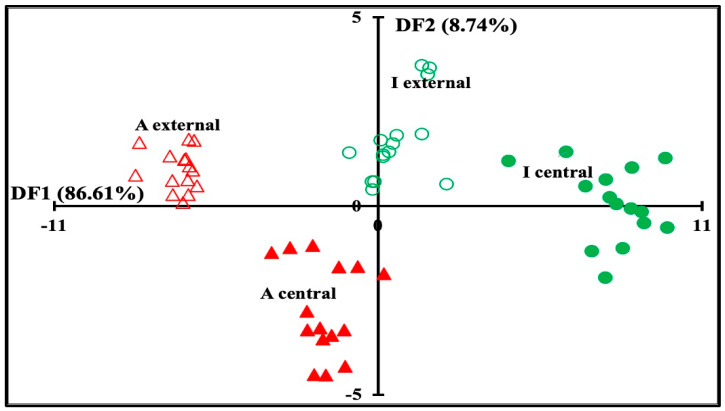
Factorial Discriminant analysis similarity map of the leave-one-out cross-validation determined by discriminant factors 1 (DF1) and 2 (DF2) of artisanal and industrial Maroilles cheeses cut at the external and central zones. Factorial discriminant analysis (FDA) was performed on the 15 concatenated PCs corresponding to PCA performed separately at 3000–2800, 1700–1500, and 1500–900 cm^−1^.

In order to take into account the whole information contained in the MIR spectral region, the concatenation approach was applied to the first five PCs of the PCA applied on each spectral region: 3000–2800, 1700–1500, and 1500–900 cm^−1^. The map of the cross-validation data set defined by the first two discriminant factors of the FDA is shown in Figure 4. Considering that discriminant factor 1 (FD1) accounted for 86.61% of the total variance, a clear discrimination between industrial and artisanal Maroilles cheeses was observed, regardless of the sampling zones. Indeed, industrial Maroilles cheeses presented mostly positive scores according to FD1, while artisanal Maroilles cheeses exhibited mostly negative scores. In addition, for the cheese type considered, a clear differentiation was noted between the external and central zones. These results suggest that concatenation of the MIR data sets may be a potential approach for recognizing Maroilles cheeses according to their type and sampling zones. Indeed, correct classification with cross-validation was observed in 96.67% of the cases (Table 4). This table shows that the external artisanal and industrial Maroilles cheeses were 100% correctly classified. Only one sample of central artisanal Maroilles cheeses was classified as belonging to external artisanal Maroilles cheeses. A similar result was noted in industrial Maroilles cheeses, since one out of 15 samples was misclassified.

### 3.5. Correlation between Physicochemical and Secondary Structure of Maroilles Cheeses

**Figure 5:**
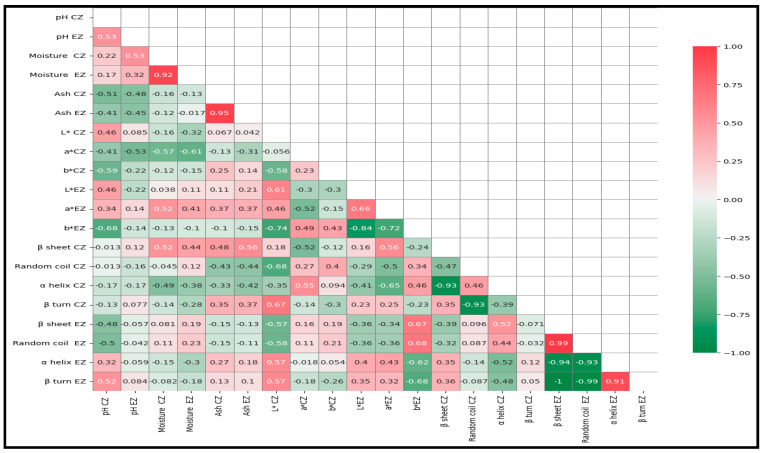
Heatmap of Pearson correlation coefficients of Maroilles cheeses obtained following physicochemical and MIR measurements (The two latter letters indicate: EZ: external zone; CZ: central zone).

A pair-wise correlation analysis was conducted to investigate the interaction between the physicochemical and structural parameters of the Maroilles cheese samples (Figure 5). Regarding the physicochemical parameters, a high positive correlation was observed for moisture and ash contents determined at the external zones (r = 0.92 and r = 0.95, respectively). For the structural parameters, the highest positive correlation was noted at the external zones for the random coil and β turn with correlation coefficients of 0.99 and 0.91, respectively. Most of the physicochemical parameters showed correlations with secondary structural parameters of Maroilles cheese samples: (i) a high positive correlation was observed between the β sheets and two physicochemical parameters determined at the central zones, including moisture (r = 0.52) and ash (r = 0.48); (ii) a significant negative correlation between the alpha helix and the moisture determined at the central zone (r = −0.49). However, only weak correlations were observed between the physicochemical and structural parameters in the external zones. We do not have an explanation for this trend, which could be due to the presence of a red ferment composed of a mixture of microorganisms (*Geotricum candidum* and *Kluvveryomyces lactis*). It has been shown that proteolytic actions bring conformational modifications in the secondary structure of proteins during cheese-making [38]. Proteolysis is influenced by environmental factors such as pH and moisture [39]. In addition, the color parameters showed some correlation with the structural parameters. For the central zone, high correlations were observed between L* and β turn (r = 0.67) and random coil (r = −0.68) and a* and α helix (r = 0.55) and β sheet (r = −0.52). For the external zone, high correlations were noted between b* and random coil (r = 0.68), β sheet (r = 0.67), β turn (r = −0.68), and α helix (r = −0.62).

## 4. Conclusions

The changes observed at the macroscopic and molecular levels of Maroilles cheese samples according to their sampling zones (external and central) and type (artisanal and industrial) have been highlighted. For instance, at the macroscopic level, physicochemical analyses showed that for a considered cheese type, artisanal and industrial Maroilles cheeses are differentiated based on fat, moisture, and b* values. At the molecular level, MIR spectral regions (3000–2800, 1700–1500, and 1500–900 cm^−1^) indicated that Maroilles cheese samples cut in the central zone presented a stronger absorbance than that those cut in the external zone. The secondary structure analysis illustrated a significant change only in the central part of Maroilles cheeses for α-helix and β-sheet levels.

The factorial discriminant analysis (FDA) applied to the first five PCs of the PCA performed on each spectral region (3000–2800, 1700–1500, and 1500–900 cm^−1^) based on the concatenation approach allowed an overall correct classification rate for the cross-validation of 96.67%.

To illustrate the relationship between the physicochemical and structural parameters of Maroilles cheeses, a heatmap was constructed, and some correlations were noted between the color, physicochemical, and structural levels determined by MIR, confirming the results obtained by FDA. It was concluded that MIR spectroscopy could be considered as a complementary technique to physicochemical techniques for the characterization of Maroilles cheeses according to the type (artisanal and industrial) and sampling zone (external and central).

However, with only 10 independent Maroilles cheese types, the current model is not yet very robust. Further analyses with more samples will be necessary to substantiate this model. This study will be extended to different Maroilles cheese types with different cheese-making procedures and different ripening stages in order to test the accuracy of the established model for recognizing Maroilles cheeses according to the type (artisanal or industrial) and sampling zones (external and central).

## Figures and Tables

**Table 1:** Investigated Maroilles cheese samples.

Brand	Producer	Label	Characteristic
Bahardes	La ferme des Bahardes	A-Bah	Length: 8 cm Width: 8 cm Weight: 180 to 200 g
Maliécourt	La ferme de Maliécourt	A-Mal	
Fleur de l’helpe	La ferme du Château Courbet	A-Fhp	
Château Courbet	La ferme du Château Courbet	A-Cct	
Moulin	La ferme du Moulin	A-Mou	
Le p’tit fromager	SARL Laiterie des étangs de Sommeron	I-Lpf	
Lesire	Ets Lesire et Roger	I-Les	
Fauquet	Les fromagers de Thiéracle	I-Fau	
Auchan	Auchan	I-Auc	
Leclerc	Leclerc	I-Lec	

In the abbreviation, the first letters –A and –I designate artisanal and industrial production, respectively.

**Table 2:** Physicochemical parameters (A) and color parameters (B) of Maroilles cheese samples.

(A)								
Cheeses	Protein (%)	Fat (%)	pH Central Zone	pH External Zone	Moisture % Central Zone	Moisture % External Zone	Ash % Central Zone	Ash % External Zone
A-Bah	19.01 ± 0.17 ^a^	26.70 ± 0.34 ^cd^	4.96 ± 0.01 ^a^	5.68 ± 0.02 ^a^	46.63 ± 0.57 ^a^	48.81 ± 0.72 ^a^	3.72 ± 0.13 ^g^	3.68 ± 0.10 ^g^
A-Mal	20.43 ± 0.35 ^b^	31.36 ± 0.14 ^g^	6.25 ± 0.02 ^f^	6.58 ± 0.11 ^d^	48.78 ± 0.02 ^b^	50.67 ± 1.05 ^b^	2.39 ± 0.03 ^a^	2.69 ± 0.04 ^ab^
A-Fhp	20.94 ± 0.10 ^bc^	28.8 ± 0.32 ^e^	5.22 ± 0.04 ^b^	5.63 ± 0.00 ^e^	50.43 ± 0.88 ^c^	53.92 ± 0.09 ^c^	2.74 ± 0.12 ^bc^	2.94 ± 0.25 ^cd^
A-Cct	21.36 ± 0.09 ^cd^	29.84 ± 0.40 ^f^	5.19 ± 0.04 ^b^	6.90 ± 0.15 ^f^	53.59 ± 0.14 ^d^	53.91 ± 0.10 ^c^	2.66 ± 0.14 ^b^	2.73 ± 0.04 ^ab^
A-Mou	19.20 ± 0.13 ^a^	27.05 ± 0.24 ^d^	5.48 ± 0.17 ^c^	6.39 ± 0.01 ^bc^	48.43 ± 0.42 ^b^	49.73 ± 0.25 ^ab^	2.57 ± 0.02 ^ah^	2.61 ± 0.05 ^a^
I-Lpf	21.74 ± 0.11 ^d^	21.85 ± 0.27 ^a^	5.82 ± 0.04 ^e^	6.74 ± 0.05 ^e^	52.76 ± 0.25 ^d^	54.76 ± 0.58 ^cd^	2.89 ± 0.00 ^cd^	3.13 ± 0.00 ^de^
I-Les	19.23 ± 0.73 ^a^	25.88 ± 0.55 ^b^	5.78 ± 0.08 ^e^	6.36 ± 0.09 ^b^	54.49 ± 0.03 ^d^	55.51± 0.08 ^de^	3.17 ± 0.02 ^ef^	3.09 ± 0.07 ^de^
I-Fau	18.94 ± 0.79 ^a^	26.06 ± 0.38 ^bc^	5.67 ± 0.05 ^dej^	6.57 ± 0.00 ^d^	55.25 ± 0.03 ^c^	56.18± 0.97 ^e^	2.68 ± 0.12 ^b^	2.82 ± 0.04 ^bc^
I-Auc	20.39 ± 0.87 ^b^	26.14 ± 0.79 ^bc^	5.55 ± 0.03 ^cd^	6.53 ± 0.04 ^cd^	56.79 ± 0.02 ^f^	62.15 ± 0.02 ^f^	3.02 ± 0.17 ^de^	3.27 ± 0.06 ^ef^
I-Lec	24.09 ± 0.08 ^e^	25.74 ± 0.25 ^b^	5.45 ± 0.00 ^c^	6.38 ± 0.00 ^bc^	50.12 ± 0.22 ^c^	50.18 ± 0.32 ^b^	3.35 ± 0.01 ^f^	3.42 ± 0.02 ^f^
Mean								
A cheeses	20.19 ± 1.04 ^a^	28.75 ± 1.94 ^b^	5.42 ± 0.50 ^a^	6.24 ± 0.57 ^a^	49.57 ± 2.62 ^a^	51.41 ± 2.38 ^a^	2.81 ± 0.52 ^a^	2.93 ± 0.44 ^a^
I cheeses	20.88 ± 2.11 ^a^	25.13 ± 1.84 ^a^	5.65 ± 0.15 ^a^	6.52 ± 0.16 ^a^	53.88 ± 2.56 ^b^	55.76 ± 4.28 ^b^	3.02 ± 0.26 ^a^	3.15 ± 0.22 ^a^
**(B)**								
**Cheeses**	**Central Zone**				**External Zone**			
	L*	a*	b*		L*	a*		b*
A-Bah	85.52 ± 0.08 ^d^	−0.63 ± 0.01 ^i^	25.66 ± 0.08 ^i^		83.93 ± 0.05 ^b^	−0.23 ± 0.02 ^g^		25.45 ± 0.04 ^e^
A-Mal	87.66 ± 0.02 ^h^	−0.85 ± 0.01 ^e^	17.32 ± 0.03 ^a^		85.00 ± 0.01 ^ef^	−0.14 ± 0.01 ^h^		22.29 ± 0.01 ^a^
A-Fhp	84.48 ± 0.03 ^b^	−1.77 ± 0.01 ^a^	22.37 ± 0.03 ^e^		84.04 ± 0.04 ^b^	−0.76 ± 0.01 ^e^		28.75 ± 0.03 ^g^
A-Cct	84.23 ± 0.07 ^a^	−0.56 ± 0.01 ^g^	23.37 ± 0.06 ^g^		79.20 ± 0.00 ^a^	−1.46 ± 0.00 ^c^		30.49 ± 0.01 ^h^
A-Mou	85.80 ± 0.08 ^e^	−0.33 ± 0.00 ^h^	23.52 ± 0.14 ^g^		84.00 ± 0.06 ^ef^	−0.55 ± 0.02 ^f^		25.69 ± 0.13 ^f^
I-Lpf	84.58 ± 0.02 ^b^	−1.37 ± 0.01 ^c^	24.69 ± 0.03 ^h^		84.32 ± 0.01 ^c^	1.06 ± 0.01 ^d^		25.00 ± 0.08 ^d^
I-Les	86.62 ± 0.02 ^f^	−0.71 ± 0.00 ^f^	21.34 ± 0.01 ^d^		84.70 ± 0.17 ^d^	2.35 ± 0.01 ^a^		22.56 ± 0.04 ^b^
I-Fau	87.39 ± 0.02 ^g^	−0.95 ± 0.02 ^d^	23.10 ± 0.06 ^f^		86.52 ± 0.02 ^g^	1.71 ± 0.01 ^b^		22.36 ± 0.02 ^a^
I-Auc	84.97 ± 0.04 ^c^	−1.72 ± 0.00 ^b^	19.68 ± 0.09 ^c^		84.87 ± 0.02 ^e^	1.07 ± 0.02 ^d^		24.11 ± 0.07 ^c^
I-Lec	88.47 ± 0.05 ^i^	−0.83± 0.00 ^e^	19.00 ± 0.01 ^b^		85.10 ± 0.03 ^f^	1.43 ± 0.01 ^c^		24.06 ± 0.03 ^c^
Mean								
A cheeses	85.54 ± 1.36 ^a^	−0.83 ± 0.56 ^a^	22.45 ± 3.11 ^a^		83.43 ± 2.42 ^a^	−0.63 ± 0.53 ^b^		26.53 ± 3.18 ^b^
I cheeses	86.40 ± 1.63 ^a^	−1.12 ± 0.42 ^a^	21.56 ± 2.36 ^a^		85.10 ± 0.84 ^a^	−1.52 ± 0.54 ^a^		23.62 ± 1.12 ^a^

Values represent means ± SD (*n* = 3). Different superscript letters indicate significant differences among Maroilles cheese types at *p* < 0.05 (Fisher test). The first letters –A and –I designate artisanal and industrial production, respectively.

**Table 3:** Secondary structure level (%) of artisanal and industrial Maroilles cheese samples.

Cheeses	Central Zone				External Zone			
	β Sheet	Random Coil Structure	α Helix	β Turn	β Sheet	Random Coil Structure	α Helix	β Turn
A-Bah	28.64 ± 0.21 ^cd^	11.53 ± 0.48 ^bcd^	14.25 ± 0.05 ^abc^	45.67 ± 0.11 ^d^	27.63 ± 0.40 ^c^	11.00 ± 0.22 ^b^	14.73 ± 0.08 ^de^	46.63 ± 0.21 ^e^
A-Mal	27.45 ± 0.26 ^b^	11.63 ± 0.50 ^bcd^	14.47 ± 0.02 ^bcd^	45.38 ± 0.13 ^c^	26.87 ± 0.15 ^a^	10.53 ± 0.17 ^a^	14.63 ± 0.04 ^d^	47.93 ± 0.14 ^g^
A-Fhp	27.49 ± 0.40 ^b^	12.11 ± 0.73 ^d^	14.73 ± 0.09 ^d^	44.86 ± 0.20 ^a^	30.00 ± 0.12 ^f^	12.20 ± 0.06 ^d^	13.93 ± 0.09 ^ab^	43.8 ± 0.07 ^a^
A-Cct	28.40 ± 0.28 ^c^	11.63 ± 0.04 ^bcd^	14.57 ± 0.06 ^cd^	45.40 ± 0.06 ^c^	29.33 ± 0.06 ^e^	11.90 ± 0.16 ^cd^	14.03 ± 0.15 ^b^	44.70 ± 0.14 ^c^
A-Mou	24.47 ± 0.43 ^a^	11.80 ± 0.18 ^cd^	15.61 ± 0.19 ^e^	45.29 ± 0.13 ^c^	30.00 ± 0.17 ^f^	12.10 ± 0.16 ^d^	14.00 ± 0.09 ^b^	43.90 ± 0.08 ^a^
I-Lpf	28.57 ± 0.29 ^cd^	11.60 ± 0.23 ^bcd^	14.10 ± 0.22 ^ab^	45.03 ± 0.05 ^ab^	28.83 ± 0.15 ^d^	11.60 ± 0.03 ^c^	14.33 ± 0.16 ^c^	45.23 ± 0.04 ^d^
I-Les	28.90 ± 0.56 ^cde^	11.43 ± 0.24 ^bc^	14.22 ± 0.37 ^abc^	44.87 ± 0.06 ^a^	27.30 ± 0.22 ^b^	10.60 ± 0.05 ^a^	14.80 ± 0.20 ^e^	47.23 ± 0.05 ^f^
I-Fau	29.47 ± 0. 35 ^f^	11.11 ± 0.28 ^b^	13.41 ± 0.36 ^a^	46.01 ± 0.22 ^e^	27.60 ± 0.09 ^bc^	11.07 ± 0.34 ^b^	14.77 ± 0.04 ^de^	46.53 ± 0.10 ^e^
I-Auc	29.10 ± 0.40 ^def^	11.30 ± 0.26 ^bc^	14.17 ± 0.09 ^abc^	45.42 ± 0.21 ^c^	29.63 ± 0.04 ^e^	12.07 ± 0.34 ^d^	13.80 ± 0.10 ^a^	44.47 ± 0.08 ^b^
I-Lec	29.33 ± 0.61 ^ef^	9.31 ± 0.11 ^a^	14.00 ± 0.03 ^a^	47.36 ± 0.15 ^f^	28.90 ± 0.15 ^d^	11.60 ± 0.06 ^c^	14.30 ± 0.15 ^c^	45.20 ± 0.05 ^d^
Mean								
A cheeses	27.29 ± 1.66 ^a^	11.74 ± 0.23 ^a^	14.73 ± 0.52 ^b^	45.32 ± 0.29 ^a^	28.77 ± 1.44 ^a^	11.55 ± 0.74 ^a^	14.26 ± 0.38 ^a^	45.39 ± 1.82 ^a^
I cheeses	29.07 ± 0.36 ^b^	10.95 ± 0.93 ^a^	13.98 ± 0.33 ^a^	45.74 ± 1.01 ^a^	28.45 ± 0.97 ^a^	11.39 ± 0.57 ^a^	14.40 ± 0.41 ^a^	45.73 ± 1.12 ^a^

Values represent means ± SD (*n* = 3), different superscript letters indicate significant differences among Maroilles cheese types at *p* < 0.05 (Fisher test). The first letters –A and –I designate artisanal and industrial production, respectively.

**Table 4:** Classification table of factorial discriminant analysis with leave-one-out cross-validation of artisanal and industrial Maroilles cheeses cut at the external and central zones.

Predicted/Observed	A Central	A External	I Central	I External	Total	% of Correct Classification
A central	14	1	0	0	15	93.33%
A external	0	15	0	0	15	100.00%
I central	0	0	14	1	15	93.33%
I external	0	0	0	15	15	100.00%
Total	14	16	14	16	60	**96.67%**

The first letters –A and –I designate artisanal and industrial production, respectively.

## Data Availability

The original contributions presented in the study are included in the article, and further inquiries can be directed to the corresponding author.
